# eGenomics: Cataloguing our Complete Genome Collection

**DOI:** 10.1002/cfg.494

**Published:** 2005

**Authors:** Dawn Field, George Garrity, Norman Morrison, Jeremy Selengut, Peter Sterk, Tatiana Tatusova, Nick Thomson

**Affiliations:** 1 Molecular Evolution and Bioinformatics Section Mansfield Road Oxford Centre for Ecology and Hydrology Oxford OX1 3SR United Kingdom; 2 Department of Microbiology and Molecular Genetics Michigan State University East Lansing MI 48824 USA; 3 School of Computer Science University of Manchester Manchester UK; 4 The NERC Environmental Bioinformatics Centre Oxford Centre for Ecology and Hydrology Oxford UK; 5 The Institute for Genomic Research 9712 Medical Center Drive Rockville MD 20850 USA; 6 EMBL Outstation — The European Bioinformatics Institute (EBI) Wellcome Trust Genome Campus Hinxton Cambridge CB10 1SD UK; 7 National Center for Biotechnology Information National Institutes of Health Bethesda MD 20894 USA; 8 The Pathogen Sequencing Unit The Wellcome Trust Sanger Institute Wellcome Trust Genome Campus Hinxton Cambridge CB10 1SA UK


**Comparative and Functional Genomics**
*Comp Funct Genom* 2005; **6**: 363–368.
Published online in Wiley InterScience (www.interscience.wiley.com). **DOI:** 10.1002/cfg.494

**Meeting Report**

### eGenomics: Cataloguing Our Complete Genome Collection

Dawn Field[1]*, George Garrity[2], Norman Morrison[3,4], Jeremy Selengut[5], Peter Sterk[6], Tatiana Tatusova[7] and Nick Thomson[8]

[1]*Molecular Evolution and Bioinformatics Section, Mansfield Road, Oxford Centre for Ecology and Hydrology, Oxford OX1 3SR, UK*
[2]*Department of Microbiology and Molecular Genetics, Michigan State University, East Lansing, MI 48824, USA*
[3]*School of Computer Science, University of Manchester, Manchester, UK*
[4]*The NERC Environmental Bioinformatics Centre, Oxford Centre for Ecology and Hydrology, Oxford, UK*
[5]*The Institute for Genomic Research, 9712 Medical Center Drive, Rockville, MD 20850, USA*
[6]*EMBL Outstation — The European Bioinformatics Institute (EBI), Wellcome Trust Genome Campus, Hinxton, Cambridge CB10 1SD, UK*
[7]*National Center for Biotechnology Information, National Institutes of Health, Bethesda, MD 20894, USA*
[8]*The Pathogen Sequencing Unit, The Wellcome Trust Sanger Institute, Wellcome Trust Genome Campus, Hinxton, Cambridge CB10 1SA, UK*

**Correspondence to:*
*Dawn Field, Molecular Evolution and Bioinformatics Section, Mansfield Road, Oxford Centre for Ecology and Hydrology, Oxford OX1 3SR, UK.*
*E-mail: dfield@ceh.ac.uk*

Received: 30 September 2005
Revised: 3 October 2005
Accepted: 4 October 2005

**Keywords:** **genomes; bioinformatics; metadata; data standards; genomic databases; computational biology; ecology; environment**

There is increasing recognition that the scientific community at large would benefit from the development of a new standard to capture a richer set of information describing complete genomes. This would ensure that those generating the genomes contributed to the quality and quantity of metadata available. Rapidly evolving high-throughput genomic sequencing technologies are generating data at an exponentially increasing rate. This poses new opportunities but also new challenges. The traditional approach to genomic sequencing has been on a 'per species' basis. However, an increasing number of 'genomes' are now being sequenced that represent not only individuals of cultivated and uncultivated organisms but also populations and communities from environmental samples. Clearly, for adequate interpretation of this type of data, simply recording only the most basic information is no longer sufficient. The time to act is now, as this deluge of data is only set to increase, especially with the emergence of ultra-high-throughput sequencing capabilities.

Community-driven standards have the best chance of success if developed within the auspices of international working groups. To discuss the need for a new genomic standard, **Dawn Field** and **Tatiana Tatusova** organized a workshop entitled 'eGenomics: Cataloguing our Complete Genome Collection' with funding from the National Institute for Environmental E-Science (NIE*e*S). This workshop took place at NIE*e*S in Cambridge, UK, on 7–9 September 2005. Participants included biologists, computer scientists, those building genomic databases and conducting large-scale comparative genomic analyses, and those with experience of building community-based standards. The outputs of the meeting include an improved discussion document describing the core pieces of information to

Copyright © 2006 John Wiley & Sons, Ltd.

be collected in such a standard (the ‘checklist’), a list of newly formed working groups whose members will work together to refine the checklist, and an open call for the active involvement of the wider community in this standardization effort.

The workshop began with a welcome from **Dawn Field**. She described that this workshop came about as a result of an opinion piece she had written with Jennifer Hughes on the benefits of developing a new genomic standard to capture a richer set of metadata about all complete genome sequences (Field and Hughes, 2005). In that piece, they suggested that such a standard could only emerge under the auspices of an international working group, and this workshop, funded by NIE*e*S, was the first step towards this goal. She thanked Tatiana Tatusova for her participation as co-organizer, as the oversight by representatives of the international sequence databases would be essential in guiding such a project. The invited speakers were brought together to help achieve three major goals at the workshop: to identify the scientific questions driving the need for more metadata; to look for ways to harmonize existing and future efforts at metadata capture; and to develop a more detailed vision of the shape a new standard might take. More specifically, it was hoped that the group would use this opportunity to discuss features for inclusion in the draft checklist, discuss potential mechanisms for capturing and exchanging metadata, and discuss how the community might organize itself to make such a standard a reality.

**Stuart Ballard** (NIE*e*S) followed up with a welcome to **NIE*e*S** [1], which is working, along with eight regional and one national eScience centres in the UK, towards the eScience vision of making computing power as readily available as power on an electrical grid. Stuart overviewed the four types of eScience technologies: computing power, data sharing, applications provision and communication. Services provided by NIE*e*S include the ability to consider proposals from the community for funding training events, workshops and working groups. NIE*e*S also runs a summer school and road shows, and invites visitors to the centre to learn about eScience. NIE*e*S serves as a first point of contact for any UK environmental eScience enquiries. Further, Stuart stressed that the current priority of the centre is to directly help environmental scientists incorporate Grid technologies into their research.

The rest of the workshop was organized into five sessions, which are described below, with the session on Day Three being devoted completely to discussion. By the end of the meeting, consensus was reached that such a standard should evolve and this group accepted responsibility for achieving this.

### Session I: Overview of our current and future genome collection

The first session was designed to set the stage for the rest of the workshop by providing a series of talks on past, current and future genome and metagenomic projects. The first speaker, **Lita Proctor** (Moore Foundation), sent apologies, but Dawn Field presented her talk. Lita’s presentation overviewed the current status of the GBMF Marine Microbial Genome Sequencing Project. The sequences from Phase One (86 genomes) will begin to be deposited in GenBank in November and proposals for Phase Two (44 genomes) are currently under consideration. When this project started, it increased the global database of marine prokaryotes approximately 10-fold. Criteria for the selection of isolates were based on microbial ecology (details posted on **www.moore.org/microgenome**). The online isolate list is notable for including such information as the geographic origin, environmental context, isolation method and primary citation for each isolate.

**Julian Parkhill** (Pathogen Sequencing Unit, Sanger Institute) provided an overview of the genome sequencing projects of the Sanger Institute’s Pathogen Sequencing Unit (PSU). The list of completed and ongoing projects includes over 30 bacterial genomes, as well as the genomes of eukaryotic protozoa, pathogenic fungi, helminths and vectors. Julian highlighted the diversity of features in these genomes and stressed that the capture of metadata describing them is becoming more important as the PSU moves towards a ‘wide and deep’ sampling strategy. The PSU proposes that sequencing animal pathogens and related non-pathogens will yield additional insights into finished human pathogens. He also discussed the important differences between the qualities of closed and draft genomes, especially those that will be generated using newly invented methods of ultra-high-throughput sequencing.

Copyright © 2006 John Wiley & Sons, Ltd. 

**Robert Feldman** (SymBio Corporation) reminded the group of the challenges and opportunities associated with using metagenomic approaches to characterize organisms from the natural environment. Given that 99% of life forms on the Earth are yet to be cultivated, metagenomics provides a way to characterize completely unknown organisms from even the most remote habitats. He described his company's involvement in a wide variety of collaborative academic projects that are using metagenomic technologies to characterize diverse systems, from the microbial genomic diversity of crenarchaeal sponge symbionts, to the endosymbiont of the giant deep-sea tube-worm, *Riftia*, to those of human gut and wound tissues.

### Session II: Databases and metadata capture efforts

This session was designed to highlight a number of database initiatives that are already capturing genomic metadata or have the mission to do so. The session also highlighted the many ways in which collections of genomes can be analysed. **Tatiana Tatusova** (NCBI) opened the session with an update on the vast resources held in the **NCBI Genome Projects Database** [2]. To deal with the issue of the need for permanent, unique IDs for each genome project, the NCBI has created the Genome Projects Database. More than 1000 genome projects have been assigned unique identifiers (ProjectIDs). All database records contain manually curated organism descriptions. For the more than 700 prokaryotic projects, organism-specific attributes (shape, motility, salinity, habitat, etc.) have been collected. These attributes are indexed and searchable in Entrez and are summarized in the Organism Infotable: **http://www.ncbi.nlm.nih.gov/genomes/lproks.cgi?view=0**. She also mentioned that the GenBank/EMBL/DDBJ collaborators meeting has already discussed a mechanism for allowing community-based efforts at capturing metadata to be integrated with primary genome annotation. This would be done by an optional extension of the 'source' tag within the annotations.

**Victor Markowitz** (Lawrence Berkeley National Laboratory) described the Integrated Microbial Community Genome (IMcG) Data Management System. This system is currently being developed as an extension of the IMG [3] system and is expected to be released by the end of 2006. This ambitious project aims to integrate high quality analysis of metagenomic datasets, from microbial communities, with vast quantities of information describing the biochemistry, physiology, ecology and evolution of isolated microbial organisms. Special importance will also be placed on capturing a range of information about each 'eco-sample', including location, temperature, pH, etc. Victor also reviewed the results of the recent 16S clone library submission survey. This survey polled the wider community on descriptors, or sequence-associated information (SAI), in particular those describing habitat type, which might be useful to add to publicly available 16S and metagenomic sequence records.

**Jeremy Selengut** (Institute for Genomic Research; TIGR) talked about the **Genome Properties** [4] project, which resides within TIGR's Comprehensive Microbial Resource (CMR). The Genome Properties database represents 'meta'-data on genome projects that have been manually curated as well as imported from external databases. These data are very useful within that system as it allows end-users to organize and discover correlations with those assertions about genome-sequenced organisms via bioinformatic analyses. The major focus of the Genome Properties system is these calculated assertions (predictions) of biological processes (pathways, systems, complexes, etc.), many of which are intimately linked with observable phenotypes that might be represented by metadata. Examples include flagella, capsules, chemotaxis, aerobic vs. anaerobic metabolic pathways and utilization of various sources of carbon and nitrogen.

**Natalia Maltsev** (Argonne National Laboratory) spoke about the **PUMA2** [5] system for high-throughput evolutionary analysis of metabolism. In her own words, PUMA2 is 'an interactive integrated environment for high-throughput genetic sequence analysis and metabolic reconstruction with a Grid-based computational back-end'. PUMA2 contains automated metabolic reconstructions for over 200 completely sequenced organisms. Natalia's group is particularly interested in populating this rich database with further ecological and phenotypic data, and she mentioned that the database was currently populated using the NCBI Genome Projects database and the

Copyright © 2006 John Wiley & Sons, Ltd. 

TIGR Genome Properties database, in addition to information manually curated from *Bergey's Manual*.

**Dawn Field** (Oxford Centre for Ecology and Hydrology) described how efforts to curate metadata describing the genomic features and ecologies of all complete genomes led to the proposal for a new genomic standard (Field and Hughes, 2005) that prompted this workshop. She reviewed some of the details of the draft checklist, in particular the types of data that might be collected, and emphasized the need for the community to be able to capture and exchange such information easily. She reported that the **GenomeMine** [6] database now supports the submission of datasets containing sets of genomic attributes. The group is currently developing the Genomic Metadata Exchange format (GnoME) as a better way of capturing and exchanging such information, especially between databases.

**Dave Ussery** (Technical University of Denmark) gave an overview of 20 different ways to analyse prokaryotic genomes. Each method, which ranged from analysis of genome length to amino acid usage to relative abundances of secretory proteins, has been previously highlighted in the 'Genome Update' column he writes for the journal *Microbiology*. Much of the data and the tools used to generate these analyses are available in the **GenomeAtlas** [7] database, which his group has built.

### Session III: Allied projects

Day Two opened with a session on allied projects. Representatives of these projects were selected both to highlight the potentially direct ways in which these projects might inform the development of a new genomic standard and to share their experiences (successes and pitfalls) of pioneering successful community-based standardization activities. **Michael Ashburner** (EBI) kicked off the session with an overview and update on the activities of the **Gene Ontology** (GO) [8] consortium. **Suzi Lewis** (University of California at Berkeley) followed this up with a very helpful overview of steps required to successfully build community-based ontologies. **Robert Stevens** (University of Manchester) spoke further on the value of face-to-face interactions when building ontologies. **Jessie Kennedy** (Napier University) discussed the problems associated with using scientific (Latin) names for referring to organisms in datasets, as they have multiple definitions and could be a source of error in analysis of data from multiple sources. A potential solution, the **Taxonomic Concept,** was described, which gives context to the meaning of names, making them unique and unambiguous [9]. **Norman Morrison** (NERC Environmental Bioinformatics Centre) discussed the development of the Env standard and its application as **MIAME/Env** [10], an extension of the MIAME standard that can now be used to describe environmental transcriptomic experiments.

**George Garrity** (Michigan State University) described his future vision for a resource that could integrate information from disparate sources using DOI technologies. Using a metadata model based on this technology, he and his collaborators are currently building a prototype application that will serve up mini-monographs in the form of free-floating information objects and persistently resolve to the correct name in a future-proof way. The model is extensible and permits incorporation of phenotypic data through the use of persistent identifiers. 'PhenBank' would bring together information from ***Bergey's Manual*** [11], the **Ribosomal Database Project** [12], taxonomic sources such as the **Taxonomic Outline of Prokaryotes** [13], publishers, genomic databases, culture collections, the primary literature and community-based datastores.

### Session IV: Case studies

These two talks brought the more abstract discussions of the previous sessions into focus with more specific descriptions of the salient features of particular groups of organisms. **Nick Thomson** (Pathogen Sequencing Unit, Sanger Institute) covered examples from the two extremes of bacterial genome evolution. *Salmonella typhi* and pathogenic *Escherichia coli* were used to illustrate the staggering degree of lateral gene transfer that has a direct impact on pathogenesis and lifestyle. Also featured were the Chlamydiae, which occupy the other end of the spectrum in terms of genome variation. Although the host-ranges and clinical manifestations of chlamydial infections are protean, their genomes are remarkably conserved, with only a small proportion of the genes being species-specific. This creates an important opportunity;

Copyright © 2006 John Wiley & Sons, Ltd. 

since the genomes are highly conserved with little variation, any difference in site of infection or clinical outcome may be related to genotype, the proviso being that this metadata is captured along with the genome sequence. In the longer term, when we sequence many more isolates of the same strain, e.g. members of the Salmonellae, we will also be able to do similar correlations, with the same caveat. **Sarah Turner** (Oxford Centre for Ecology and Hydrology) talked about non-autonomously replicating genomes of plasmids and viruses. She described the salient features of the genomes of the pQBR environmental plasmids, baculoviruses and rabbit haemorrhagic disease viruses, all of which had been used as case studies to develop the list of attributes in the draft checklist. The take-home message of her talk was that the 'environmental context' of such genomes is the host and that without phenotypic and geographic information (epidemiological information in the case of viruses) their study is severely limited.

**Matt Kane** (National Science Foundation; NSF) closed this session, and the formal presentations of the workshop, by re-emphasizing the importance of understanding the microbial world and the need for the right tools and resources to do so. Quantitative data from a recent study, which examined the geographic and habitat sources of environmental isolates in the American Type Culture Collection (ATCC; Floyd *et al.* 2005), was used to illustrate that not only have very few microbes been captured in the laboratory, but those that have constitute a very biased sample that virtually ignores traditional biodiversity hotspots, such as the Amazon basin. Kane closed his talk by describing NSF's diverse portfolio of research in microbial biology. The most relevant programmes supporting microbial research are the Microbial Genome Sequencing Program, Microbial Observatories and Microbial Interactions and Processes, and Prokaryotic Molecular and Cellular Biology. Other programmes of direct relevance include the Biological Oceanography and Assembling the Tree of Life programme areas, as well as an anticipated Environmental Genomics activity.

### Summary

The sessions of this workshop moved participant discussions from a broad view of the value and diversity of our current genome collection, to projects that are working to collect, present and analyse information from these genomes, to examples of how other communities have successfully driven standards development, to the specific consideration of the salient features of particular genomes. Speakers stimulated lively discussions throughout the workshop and most talks ran longer than expected because of probing questions from the audience.

The projects described highlighted the diversity of taxa for which we have complete genomes and the growing importance of environmental and metagenomic data. They also highlighted that a number of initiatives are improving the quality of genomic descriptions available, but there is still a demand for curated information, which is best provided by those responsible for generating a given genome. The talks in the 'allied projects' session showed the group the benefits of learning from the experiences of other communities. The successful launch of a new genomic standard will take not only patience and enthusiasm from an international group of participants, but repeated meetings and funding.

There was a strong sense of a shared mission among participants and an eagerness to offer solutions to ongoing issues in the area of metadata capture and presentation. For example, Matt Kane stressed in his talk that there is a considerable amount of environmental genetic data already in GenBank from targeted gene surveys, but the corresponding physicochemical and biogeochemical data can only be found by looking in the primary literature. Data from environmental genetic surveys requires the same metadata needed to maximize the usefulness of metagenomic information. Tatiana Tatusova of the NCBI volunteered to look into whether, as a first step towards this goal, sequences in GenBank that have been recovered directly from the environment could be logically grouped together by publication and in the future be tagged with additional metadata.

The group expressed a shared vision of a future where it would be routine to explore relationships between genomes, not only by taxonomy or traditional features such as shared proteins, but also by a wide variety of phenotypic, physiological, ecological or environmental attributes. Further, the group was extremely interested in the ability to harvest such data for the sake of populating

Copyright © 2006 John Wiley & Sons, Ltd.

their own databases and for large-scale comparative genomic analyses. *Bergey's Manual* was repeatedly mentioned as the preferred, definitive source of information about prokaryotic biology and taxonomy. There was much discussion and sharing of frustrations at the time and effort it took to curate even small amounts of information out of *Bergey's Manual* or the primary literature, despite the obvious value of having such organismal information tagged to genomic information. There was interest in the possibility of seeing the information in *Bergey's Manual* publicly available in electronic format. It was repeatedly mentioned that there is a need to harmonize across efforts, for example to work together to better distribute datasets and metadata, and there was special interest in the ability to share genomic annotations.

It was hoped from the start of this workshop that this group could come together to form a formal international consortium. This happened during the discussion sessions of Day Three, when participants of the workshop agreed to work together through virtual communication and future meetings towards recommendations on the type of information to capture, ways in which data can be more easily exchanged, and an implementation. Funding for future workshops has been secured in part in the form of a NERC International Opportunities Fund Award to DF (NE/3 521 773/1). The group is making an open call for new members to join the identified working groups dedicated to descriptions of different taxa (metagenomes, eukaryotes, prokaryotes, viruses, plasmids, and organelles) and concepts. Plans for future meetings and activities will be posted on the 'Cataloging our Current Genome Collection' website (**http://www.genomics.ceh.ac.uk/genomecatalogue/**). The draft checklist will also be available for community feedback. Anyone interested in knowing more about, or joining, this effort is encouraged to contact us.

### Acknowledgements

The authors acknowledge the invaluable contributions of all of the participants who attended the workshop. This workshop was funded by the National Institute for Environmental eScience (NIEeS) and the 'Cataloguing our Complete Genome Collection' project is supported by a NERC International Opportunities Fund Award (NE/3521773/1) to D.F.

### URLs

[1] The National Institute for Environmental eScience: **http://www.niees.ac.uk/**
[2] NCBI Genome Projects Database: **http://www.ncbi.nlm.nih.gov/entrez/query.fcgi?db=genomeprj**
[3] Integrated Microbial Genomes (IMG): **http://img.jgi.doe.gov/pub/main.cgi**
[4] Genome Properties Database: **http://www.tigr.org/tigr-scripts/CMR2/genome_properties**
[5] PUMA2: **http://compbio.mcs.anl.gov/puma2/cgi-bin/index.cgi**
[6] GenomeMine: **http://www.genomics.ceh.ac.uk/GMINE/**
[7] GenomeAtlas: **http://www.cbs.dtu.dk/services/GenomeAtlas/**
[8] Gene Ontology Consortium: **http://www.geneontology.org/**
[9] Taxonomic Concept: **http://www.soc.napier.ac.uk/tdwg/**
[10] MIAME/Env: **http://envgen.nox.ac.uk/miame/miame_env.html**
[11] *Bergey's Manual* Trust: **http://www.bergeys.org**
[12] The Ribosomal Database: **http://rdp.cme.msu.edu**
[13] The Taxonomic Outline of the Prokaryotes: **http://www.bergeysoutline.com**

### References

Field D, Hughes J. 2005. Cataloguing our current genome collection. *Microbiology* **151**: 1016–1019.

Floyd MM, Tang J, Kane M, *et al*. 2005. Captured diversity in a culture collection: case study of the geographic and habitat distributions of environmental isolates held at the American Type Culture Collection. *Appl Environ Microbiol* **71**: 2813–2823.

Copyright © 2006 John Wiley & Sons, Ltd.
